# Down-Regulation of Cytokinin Receptor Gene *SlHK2* Improves Plant Tolerance to Drought, Heat, and Combined Stresses in Tomato

**DOI:** 10.3390/plants11020154

**Published:** 2022-01-07

**Authors:** Naveed Mushtaq, Yong Wang, Junmiao Fan, Yi Li, Jing Ding

**Affiliations:** 1 State Key Laboratory of Crop Genetics and Germplasm Enhancement, College of Horticulture, Nanjing Agricultural University, Nanjing 210095, China; naveedmushtaq001@gmail.com (N.M.); yongwang@henau.edu.com (Y.W.); 2018204022@njau.edu.cn (J.F.); yi.li@uconn.edu (Y.L.); 2 College of Horticulture, Henan Agricultural University, 63 Nongye Road, Zhengzhou 450002, China; 3 Department of Plant Science and Landscape Architecture, University of Connecticut, Storrs, CT 06269, USA

**Keywords:** abiotic stress, drought stress, heat stress, combined stress, photosynthesis, antioxidants, cytokinin, tomato

## Abstract

Environmental stresses negatively affect the growth and development of plants. Several previous studies have elucidated the response mechanisms of plants to drought and heat applied separately; however, these two abiotic stresses often coincide in environmental conditions. The global climate change pattern has projected that combined drought and heat stresses will tend to increase in the near future. In this study, we down-regulated the expression of a cytokinin receptor gene *SlHK2* using RNAi and investigated the role of this gene in regulating plant responses to individual drought, heat, and combined stresses (drought + heat) in tomato. Compared to the wild-type (WT), *SlHK2* RNAi plants exhibited fewer stress symptoms in response to individual and combined stress treatments. The enhanced abiotic stress tolerance of *SlHK2* RNAi plants can be associated with increased membrane stability, osmoprotectant accumulation, and antioxidant enzyme activities. Furthermore, photosynthesis machinery was also protected in *SlHK2* RNAi plants. Collectively, our results show that down-regulation of the cytokinin receptor gene *SlHK2*, and consequently cytokinin signaling, can improve plant tolerance to drought, heat, and combined stress.

## 1. Introduction

Abiotic stresses induced by environmental conditions may adversely limit plant growth and productivity [1,2]. Plants counter abiotic stresses with several changes in their morphological, biochemical, physiological, and molecular levels [3,4]. Various abiotic stresses naturally coincide in the field, where plants are exposed to multiple abiotic stresses. Among these stresses, heat and drought are significant constraints on global plant growth and food security [5,6].

Tomato is one of the most important vegetables worldwide; it often confronts a combination of stresses during its cultivation [7]. As heat and drought stresses are involved in combination, the plant’s physiological responses were notable to be inferred from individual stress analysis. The combined stress reduces yield more than a single stress; thus, the combination of drought and heat is perceived as the most severe challenge, negatively impacting plant growth and yield [1,8].

In the abiotic stress environment, relative water percentage is a critical sign of water level in plants. Tolerant plants exhibit retention of relative water content under dehydration and heat stress [9]. Moreover, malondialdehyde content (MDA) manifests a reflection of membrane degradation in plants. The abiotic stresses elevate MDA content in plants, and low concentrations of MDA have been connected with plants’ abiotic stress tolerance [10].

Photosystem II (PSII) in leaves of plants is generally sensitive in abiotic stress environments. Several previous studies have demonstrated that the combination of drought stress and heat stress severely impairs the activity of PSII in plants, and reduces the electron transport rate and quantum yield of PSII. Chlorophyll fluorescence analysis is a reliable and non-destructive method of determining PSII machinery impairment in abiotic stress environments [11]. Along with damage to photosynthetic activities, drought and heat stresses repress important enzymes and impact membrane stability, prompting metabolic instability. Plants confront the consequences of these stresses via the accumulation of osmoprotectants. Upon exposure to individual and combined stresses, plants commonly acquire three types of osmoprotectants: sugars, betaines, and proline. Apart from its osmoprotectant role, proline displays many other protecting effects, together with radical scavenging and maintenance of redox balance [12].

In abiotic stress tolerance, antioxidant protection systems perform an essential function in plants by alleviating oxidative stress via detoxifying reactive oxygen species (ROS). Such antioxidants (enzymatic systems) include peroxidase (POD), glutathione reductase (GR), superoxide dismutase (SOD), catalase (CAT), ascorbate peroxidase (APX), and dehydroascorbate reductase (DHAR) [13]. The tolerant plants displayed high antioxidant enzyme activity, collectively with more leading CAT and SOD activities [14]. The ascorbate-glutathione pathway (which includes APX and GR) confers the prime H_2_O_2_-detoxification mechanism in plants. The activities of enzymes DHAR, APX, DHAR exhibit a significant enhancement in plants exposed to abiotic stresses [10,13].

Cytokinins were initially distinguished as factors that can improve the proliferation of cells [15]. Subsequently, this type of phytohormone was linked to various perspectives regarding plants’ biological functions, together with the germination of seeds, vasculature development, flowering, meristem activities, and regulating leaf aging [16]. Various stresses, such as drought and salt, reduce cytokinin levels in plants by suppressing cytokinin signaling genes. A lowered cytokinin level mainly enhances sensitivity towards ABA, which is an imperative response to tolerating stress conditions [17,18]. In transgenic tobacco Pssu-*ipt* plants, cytokinins increased the activity of antioxidant enzymes, including GR, SOD, APX, and GSH [19,20]. Cytokinin signaling in plants uses a multistep phosphorelay system involving kinases, histidine phosphotransfer proteins, and response regulators [21]. In *Arabidopsis*, *AHK4*, *AHK3*, and *AHK2* have been recognized as cytokinin receptors for cytokinin signaling [22]. The cytokinin receptors *AHK2* and *AHK3* have exhibited roles in controlling organ development and different regulative functions in stems and roots. Several analyses have indicated that *AHK2* shows a negative function in plants under dehydration stress [23]. Nevertheless, the particular functions of the tomato histidine kinase 2 (*SlHK2)* during the plant’s response to abiotic stresses (drought, heat, and combined stresses) remain unclear and have not yet been studied.

In this study, we obtained *SlHK2* RNAi plants to characterize the functions of *SlHK2* in regulating plant responses to drought, heat, and combined stresses. Furthermore, we found that the maintenance of cell membrane stability, photosynthesis machinery protection, accumulation of osmoprotectants, reduced ROS accumulation, increased antioxidant enzyme activities, up-regulation of antioxidant genes, and up-regulation of cuticle wax biosynthesis genes played roles in the abiotic stress tolerance of *SlHK2* RNAi plants. Our results demonstrate that *SlHK2* acts as a negative regulator of drought, heat, and combined stresses in tomato plants.

## 2. Materials and the Methods

### 2.1. Plasmid Construction and Tomato Transformation

A 234-bp sequence specific to *SlHK2* CDS was designed for silencing *SlHK2*. The sense and antisense-strand fragments were amplified by employing the primers shown in the Supplementary Materials, Table S1; furthermore, they were cloned within the 2300GNR vector and driven by a CaMV35S promoter. The resulting plasmid p35S:SlHK2-RNAi was confirmed by PCR and sequencing before transformation. The schematic representation of this plasmid construct of *SlHK2* in tomato plants is shown in the Supplementary Materials, Figure S1. The *SlHK2* (Solyc07g047770) and reference gene *SlActin* (Solyc11g005330) sequences can be found on the tomato genome database (http://solgenomics.net/ accessed on 1 January 2020). The reporter gene *GUS* was included with the plasmid p35S:SlHK2-RNAi, under the control of the CaMV35S promoter (Figure S1). Therefore, GUS activity served as a tissue-specific indicator of the presence and expression of RNAi in transformed tomato plants. The tomato (*Solanum Lycopersicon*) cv. “Micro-Tom” plants were genetically transformed using the cotyledon explant transformation method [24]. The *SlHK2* RNAi plants rooted in 50 mg L^−1^ of kanamycin medium were moved into soil and grown in a greenhouse until maturity. The seeds were then obtained, and independent T_1_ lines were further grown to collect seeds for homozygous T_2_ lines.

### 2.2. Plant Materials and Growth Conditions

Two individual homozygous lines from the T_3_ *SlHK2* RNAi progeny, with the lowest transcript level of the endogenous *SlHK2* gene based on qPCR analysis, were chosen for research. The *SlHK2* RNAi and wild-type (WT) plants were planted in trays. After 18 days, the young seedlings were shifted into pots (11 cm diameter, 9 cm height). The seedlings were grown in greenhouse conditions (with 16 h day and 8 h night duration, along with 25 °C day and 18 °C night temperatures, 70 percent moisture content, and 250 μmol m^−2^ s^−1^ luminous intensity).

After 25 days in the greenhouse, the plants were shifted into controlled chambers (MB-teknik, Brøndby, Denmark). The chambers’ environmental parameter settings were 26 °C in the day and 20 °C at night, with 65% relative humidity, 310 ± 20 μmol m^–2^ s^–1^ PPFD, and a 13 h day. The soil was saturated daily with a solution of 1 g per liter of Murashige and Skoog (MS). The 35-day-old WT and *SlHK2 RNAi* plants were distributed in four groups (with 16 plants per cultivar for each treatment), with the following stress conditions: (1) a well-watered condition, with 26 °C day and 20 °C night temperatures and one watering daily; (2) a drought stress condition, with 26 °C day and 20 °C night temperatures and no watering for five days; (3) a heat stress condition, with 32 °C day and 26 °C night temperatures and one watering daily; and (4) a combined stress condition (drought and heat) with 32 °C day and 26 °C night temperatures and no watering. All four stress conditions were continued for five consecutive days as the WT plants exhibited phenotypic changes, such as leaf wilting on the fourth day during the pilot experiment.

### 2.3. Gas Exchange and Chlorophyll Fluorescence Parameters

The photosynthesis rate and stomatal conductance were measured via a portable photosynthesis system (LI-6400; LI-COR, Lincoln, NE, USA). Chlorophyll fluorescence analysis was conducted on the completely developed leaves of WT and *SlHK2* RNAi plants under each stress condition. During quenching measurements, the plants were adapted to a dark place for at least 22 min. Fv/Fm (maximal efficiency of PSII) was measured with a MINI-PAM fluorometer (Walz, Effeltrich, Germany) and the win control program. The other parameters, such as NPQ (non-photochemical quenching), ETR (electron transport rate), ΦPSII (quantum yield of PSII), and qL (fraction of open PSII centers) were held constant following PPFD of 300 μmol m^−2^ s^−1^ via an exterior light (Schott KL 1500, Göttingen, Germany) [11].

### 2.4. Determination of Osmoprotectant Accumulation

The proline and glycine betaine (GB) concentrations were evaluated, as explained by Grieve et al. [25]. The proline level was determined based on absorbance at 500 nm; for the GB concentration, the absorbance was measured at 360 nm. The soluble sugars were estimated from the leaves, as described by Blunden et al. [26]. The ground leaf samples (60 mg) were dissolved in 10 mL of a 55% aqueous methanol solution. For protein precipitation, 6 mL of perchloric acid were added to the samples of soluble sugars. The starch concentration in the plants’ leaves was calculated, following the procedure of Rasmussen et al. [27]. The trehalose content in the leaves was estimated via the protocol described by Schulze et al. [28]. The leaves’ samples were normalized in 5 mL of 20 mM phosphate-buffered saline (PBS) with 5.5 pH to measure trehalose contents.

### 2.5. Transcript Quantification

The leaves’ samples were collected for RNA extraction with the RNAiso Plus kit (Takara) after five days in each stress condition. For tissue-specific GUS staining, samples were taken from 55 day-old *SlHK2* RNAi plant leaves, whereas the sepal and fruit samples were collected after 20 days of DAF.

Next, the extracted RNA was converted into cDNA, following the instructions of the Prime Script™ RT reagent Kit (Takara). The DNA was removed in the samples via a gDNA eraser present inside the kits. The synthesized cDNA was diluted, and qPCR analysis was performed in a CFX96™ Real-Time System (C1000™ Thermal Cycler, Bio-Rad, Hercules, CA, USA), using SYBR green supermix (Takara). *SlActin* was applied as an endogenous control, and every sample was tested in triplicate. The transcript expression levels in the plants were determined by the 2^−ΔCT^ method [1]. The primer sequences employed in the studies are shown in the Supplementary Materials, Table S1.

### 2.6. Enzymatic Activities

For the enzyme activities, 400 mg of leaves were taken from six individual plants of both WT and *SlHK2* during each stress condition (drought, heat, and combined stresses). Catalase (CAT) antioxidant activity was calculated by following the protocol outlined by Havir et al. [29]. The ascorbate peroxidase (APX) activity was measured in the leaves, as proposed by Rao et al. [30,31]. Superoxide dismutase (SOD) activity was assessed by following the method of Giannopolitis et al. [32]. Peroxidase (POD) activity was measured according to the method of Upadhyaya et al. [33]. Dehydroascorbate reductase activity (DHAR) was determined for approximately 3 min at 265 nm, following the absorbance difference subsequent to the generation of AsA [30]. According to this method, the GR activity was estimated at 340 nm by observing NADPH’s oxidation for approximately 180 s [31].

### 2.7. H_2_O_2_ Content and Lipid Peroxidation

The H_2_O_2_ content in the samples was estimated, as previously illustrated [34]. One mL of the obtained extract was added to 220 mL of 0.09% titanium dioxide with approximately 15% (*v*/*v*) H_2_SO_4_; the mixture was then centrifuged at 5000× *g* for nearly 17 min. At 420 nm, the intensity of the supernatant was examined. The H_2_O_2_ accumulation was revealed as µmol g^−1^ DW. The malondialdehyde (MDA) content was examined in the tomato leaves according to the protocol outlined by Rao et al. [29]. The leaves were normalized in 10 mL of trichloroacetic acid (TCA) liquid, for the measurement of MDA contents.

### 2.8. DAB Staining for H_2_O_2_

For the visual estimation of H_2_O_2_, DAB staining was performed via an adaptation of the protocol of Thordal-Christensen et al. [35,36]. In a DAB 1 mg mL^−1^ solution, the separated leaves were immersed at a temperature of approximately 25 °C. The process was continued for about 7 h after the leaves’ immersion, while a brown precipitate began to be evident in the leaves. Next, the leaves were heated for approximately 15 min in the ethanol, acetic acid, and glycerol solution. A bleaching solution was added in the tubes, and the leaves were incubated until the chlorophyll color was effectively removed. Pictures were taken to measure the H_2_O_2_ in the leaves.

### 2.9. Accumulation of Cuticular Wax

The total wax load was investigated in the *SlHK2* RNAi and WT unstressed plants, according to the protocol outlined by Wang et al. [37]. The analysis was conducted via gas chromatography equipped with mass spectrometry (GC-MS) and gas chromatography with flame ionization detection (GC-FID). After the sample collection, the leaves were immersed for 1 min in chloroform for extraction of the wax. After extraction, the wax samples were further dehydrated and derivative with equivalent amounts of *N*, *O*-trimethylsilyl (BSTFA), and pyridine, for 40 min at 70 °C. Next, the samples were dried in nitrogen gas and immersed in the chloroform. As the internal standard, n-tetracosane (C24) was included in the samples. The gas chromatography was equipped with the mass spectrometry (GC-MS) to quantify the wax compounds in the leaves. The wax content was measured, using the peak area in association with the C24 internal standard.

### 2.10. Relative Water Content

The leaves’ relative water content (RWC) was estimated by applying the method described by Nishiyama et al. [38]. After five days, the collected leaves of WT and *SlHK2* RNAi plants were weighed. Then, the leaf samples were moved to 50 mL tubes and hydrated for 10 h. The leaves were separated from the water, dried with the help of filter paper, and then weighed again to determine the turgid weight (TW). The dry weight (DW) was then measured by dehydrating the leaves in an oven for 48 h at 68 °C. The relative water content (RWC) was determined via the following formula:

RWC (–) = [(W − DW)/(TW − DW)] × 100

### 2.11. Electrolyte Leakage in Plants

The electrolyte leakage percentage from the collected leaves was estimated by following the protocol of Nishiyama et al. [38]. The leaves were moved to 50 mL tubes with 40 mL of sterilized water and shaken for 2.5 h. The electrolyte leakage percentage was measured according to the electric conductivity percentage at the initial boiling and subsequent boiling of the separated leaves. 

### 2.12. Statistical Analysis

Statistical analyses were completed using the simple analysis of variance (ANOVA) supported by LSD (least significant difference). In the data, differences were recognized as being significant if *p* < 0.05. The heat map was generated with the help of Heml Ink software.

## 3. Results

### 3.1. SlHK2 RNAi Plants Are More Tolerant to Drought, Heat, and Combined Stresses than WT Plants

**Figure 1:**
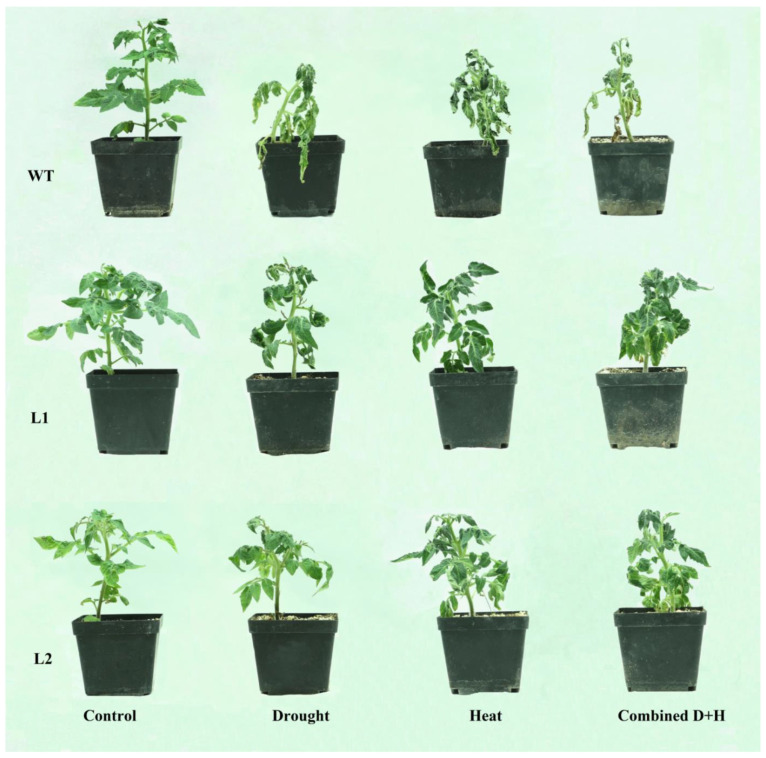
Phenotypes of the WT and *SlHK2* RNAi (**L1** and **L2**) lines under well-watered (control) and drought, heat, and combined stress conditions. The 5-week-old *SlHK2* RNAi and WT plants grown under well-watered conditions are used for treatment. Drought: 5 days of no watering; heat: 5 days of 32/26 °C (day/night) with watering once every day; combined D + H: 5 days of no watering and 32/26 °C (day/night).

Under normal conditions, *SlHK2* RNAi plants have more root length compared to WT plants (Supplementary Materials, Table S2). The *SlHK2* RNAi and WT tomato plants were subjected to five-day drought, heat, and combined stress treatments (Figure 1). Both *SlHK2* RNAi and WT plants displayed wilted leaves after those treatments. However, the combined stress’s impact was more severe than either of the individual stresses. Interestingly, the leaflets of *SlHK2* RNAi plants showed slight wilting in certain drought, heat, and combined stress treatments, while the leaflets of WT plants showed more wilting under the same treatments.

Two individual homozygous lines from the T_3_ *SlHK2* RNAi progeny (*SlHK2* L1 and *SlHK2* L2) showed a reduction in the transcript level of the *SlHK2* gene and were selected for further experiments (Figure S2). We investigated the expression of cytokinin-induced genes (*SlRR1*, *SlRR3,* and *SlRR5*) in *SlHK2* RNAi and WT plants. In control conditions, the expression level of *SlRR1*, *SlRR3*, and *SlRR5* was notably down-regulated in *SlHK2* RNAi plants when compared with WT plants. These results strengthen the successful knockdown of the receptor gene *SlHK2* in the two selected *SlHK2* RNAi lines. GUS expression was detected in various tissues of *SlHK2* RNAi plants, with histochemical assay (Figure S3). The reporter gene *GUS* was included in the plasmid p35S:SlHK2-RNAi, under the control of a CaMV35S promoter (Figure S1). Therefore, GUS activity served as a tissue specific indicator for the presence and expression of RNAi in transformed tomato plants. Our results also indicated that the GUS activity pattern of *SlHK2* may depend on the tomato plant’s developmental stages.

**Figure 2:**
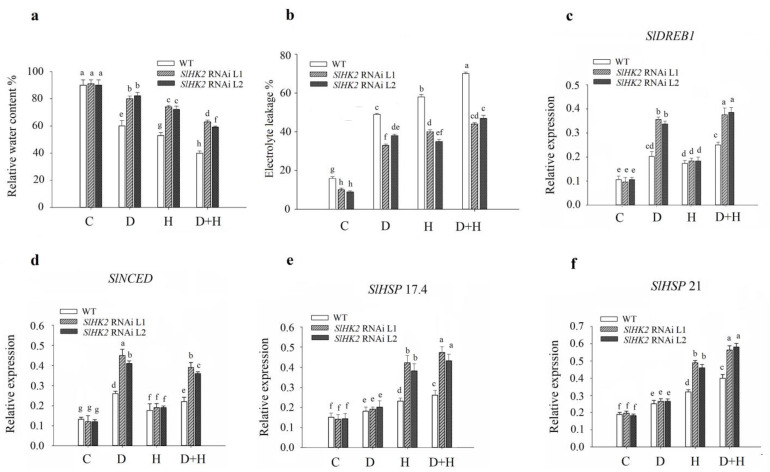
(**a**) Relative water content %, (**b**) electrolyte leakage %, transcript levels of (**c**) *SlDREB1*, (**d**) *SlNCED*, (**e**) *SlHSP 17.4*, and (**f**) *SlHSP 21*, in leaves of WT and *SlHK2* RNAi plants grown under well-watered (C), drought (D), heat (H), and combined stress (D + H) conditions. Values represent mean ± SE (n = 6). The relative expression levels were determined by quantitative real-time PCR using the *SlActin* gene as reference. Different letters indicate significant differences at *p* < 0.05 among treatments.

Moreover, we measured the relative water content (RWC) in the *SlHK2* RNAi and WT plants under single conditions of drought stress and heat stress, and under combined stress (Figure 2a). The *SlHK2* RNAi plants retained a significantly higher percentage of RWC than did the WT plants in drought stress and heat stress conditions. Compared to *SlHK2* RNAi plants, the combined stress treatment caused a severe reduction of RWC in WT plants. These results suggested that *SlHK2* RNAi plants exhibit more tolerance to drought, heat, and combined stresses. 

An electrolyte leakage study was conducted to evaluate cell membrane integrity changes after exposure to abiotic stress in *SlHK2* RNAi and WT plants. Under well-watered (control) conditions, electrolyte leakage was significantly different among *SlHK2* RNAi and WT plants. The *SlHK2* RNAi plants showed substantially lower electrolyte leakage than WT under all stress conditions (Figure 2b).

We conducted qRT-PCR validation to investigate expressional changes of drought- and heat-responsive genes in WT and *SlHK2* RNAi plants, including *SlDREB1*, *SlNCED*, *SlHSP 17.4*, and *SlHSP 21.* In drought and combined stress treatments, the expression level of *SlDREB1* and *SlNCED* was induced in both *SlHK2* RNAi and WT plants, compared to well-watered plants (Figure 2c,d). Interestingly, the expression level of *SlDREB1* and *SlNCED* was much higher (approximately 1.5-fold) in the *SlHK2* RNAi lines than in the WT plants under the drought and combined stresses. Moreover, the expression of *SlHSP 17.4* and *SlHSP 21* was significantly up-regulated in *SlHK2* plants under heat and combined stresses, compared with WT plants (Figure 2e,f). However, *SlHSP 17.4* and *SlHSP 21* showed no significant differences in *SlHK2* RNAi and WT plants under drought stress.

**Figure 3:**
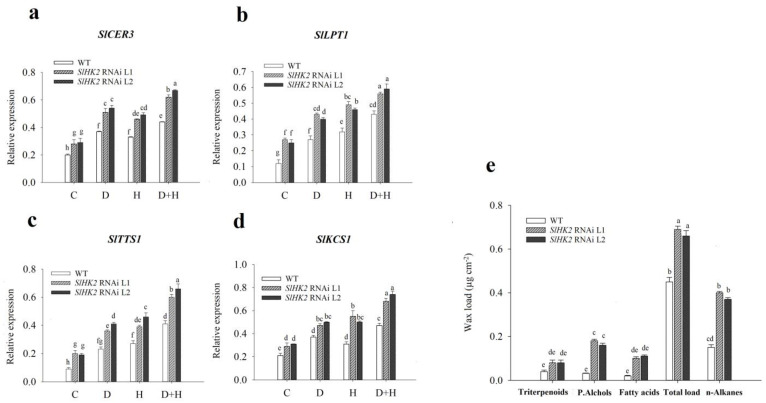
Transcript levels of (**a**) *SlCER3,* (**b**) *SlLPT1*, (**c**) *SlTTS1*, (**d**) *SlKCS1*, and (**e**) cuticular wax accumulation, in leaves of WT and *SlHK2* RNAi plants. For the quantitative real-time PCR analysis, the plants were used after five days of well-watered (C), drought (D), heat (H), and combined stress (D + H) conditions. The relative expression levels were determined by quantitative real-time PCR using the *SlActin* gene as reference. The well-watered *SlHK2* RNAi and WT plants were used to determine cuticular waxes. Cuticular waxes were extracted and analyzed using GC-MS. Values represent mean ± SE (n = 6). Different letters indicate significant differences among treatments at *p* < 0.05.

We measured the total wax loads in *SlHK2* RNAi and WT plants under normal conditions. The results showed that the total wax load in *SlHK2* RNAi was significantly higher than in WT tomato plants under controlled conditions (Figure 3e). Furthermore, we analyzed the expression of four wax biosynthesis genes, *SlCER3, SlLPT1, SlTTS1*, and *SlKCS1*, in the *SlHK2* RNAi and WT plants. The function of these genes involved alkane synthesis (*SlCER3*), lipid transportation (*SlLTP1*), triterpenoids development (*SlTTS1*), and fatty acids elongation (*SlKCS1*) [39]. Under the well-watered conditions, *SlHK2* RNAi showed a higher expression level of these genes than did the WT plants. The expression levels of *SlCER3, SlLPT1, SlTTS1*, and *SlKCS1* were induced in the *SlHK2* RNAi plants after five days of single and combined stresses (Figure 3a–d). The results also revealed that the combination of drought and heat stresses was accompanied by a higher expression level of these cuticular wax genes, in comparison to single stresses. The significant accumulation of wax loads and wax-related genes found in the *SlHK2* RNAi plants demonstrated the involvement of *SlHK2* in producing cuticular wax.

### 3.2. Photosynthesis and Gas Exchange of SlHK2 RNAi and WT Plants under Stress

**Figure 4:**
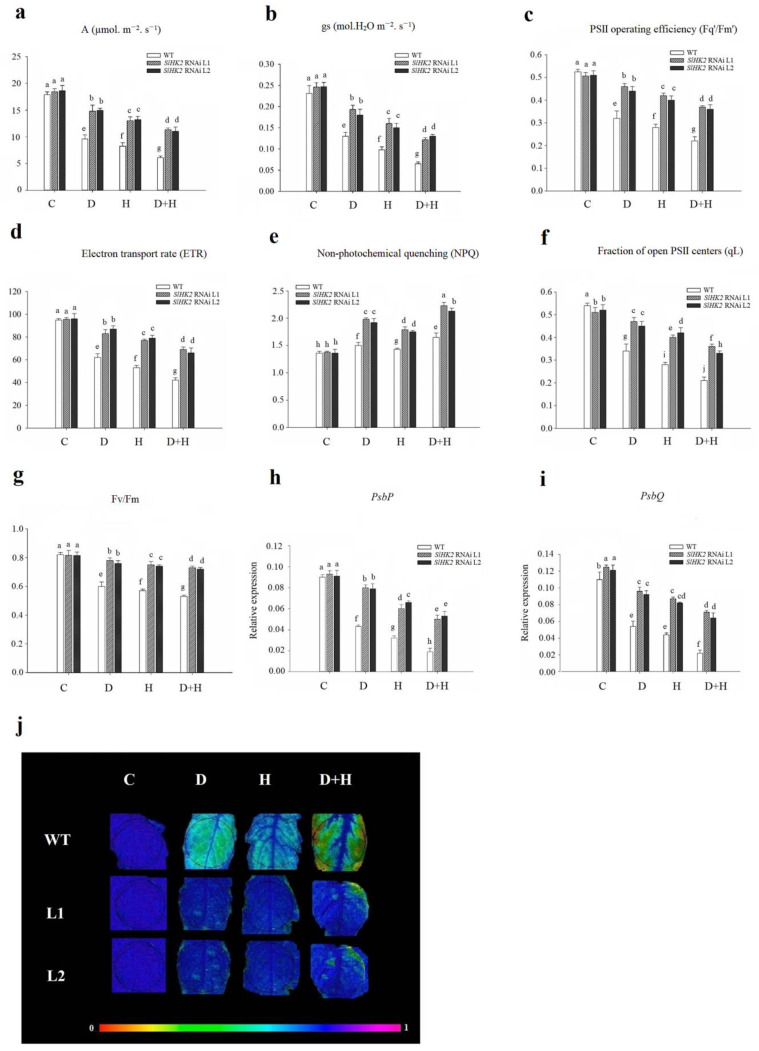
(**a**) Photosynthetic rate (A, in µmol m^−2^s^−1^), (**b**) stomatal conductance (gs, in µmol H_2_O m^−2^s^−1^), (**c**) PSII operating efficiency (Fq’/Fm’), (**d**) electron transport rate (ETR), (**e**) non-photochemical quenching (NPQ), (**f**) the fraction of open PSII centers (qL), (**g**) maximum photochemical yield of photosystem II (Fv/Fm), (**h**) transcript levels of the photosystem II oxygen-evolving complex protein gene (*PsbP*), (**i**) photosystem II oxygen-evolving complex protein gene (*PsbQ*), and (**j**) chlorophyll fluorescence imaging (Fv/Fm), in leaves of WT and *SlHK2* RNAi lines (L1 and L2). The color range shows the plants’ values from black (minimum) to pink (maximum) in chlorophyll fluorescence imaging. Spots on the leaf circle display areas damaged by these stresses in (**j**). The relative expression levels were determined by quantitative real-time PCR (using *SlActin* gene as reference). Different letters show significant differences among treatments at *p* < 0.05.

The photosynthesis rate and stomatal conductance were measured immediately after the drought, heat, and combined treatments (Figure 4). Stomatal conductance and photosynthetic activity in both *SlHK2* RNAi and WT plants were reduced in all stress conditions. Nevertheless, the decrease in these parameters was more severe in combined stresses than in either drought and heat stress independently. The reduction in the photosynthesis rate and stomatal conductance in WT was more significant under drought, heat, and combined stresses. Overall, *SlHK2* RNAi plants exhibited more remarkable photosynthetic ability when compared with WT plants.

To discover the physiological response of *SlHK2* RNAi and WT plants under independent drought, heat, and combined stress treatments, we analyzed the maximum quantum efficiency (Fv/Fm), the fraction of open PSII centers (qL), the electron transport rate (ETR), non-photochemical quenching (NPQ), and PSII operating efficiency (Fq’/Fm’) (Figure 4). Under well-watered (control) conditions, Fv/Fm, Fq’/Fm’, and ETR were not significantly different for *SlHK2* RNAi and WT plants. However, qL values were less in *SlHK2* RNAi lines than they were in WT plants. Furthermore, our results reveal that combined stress conditions have a more significant impact on tomato plants than each of the other stresses imposed individually. Comparing *SlHK2* RNAi plants with WT plants, our results show the most significant reduction of these parameters in WT plants under the combined stress treatment (Figure 4). The chlorophyll fluorescence imaging technique showed that Fv/Fm values significantly diminished in WT leaves in drought, heat, and combined stress treatments (Figure 4j). The color range shows the plants’ values, from black (minimum) to pink (maximum). Spots on the leaf circle display areas that were damaged by these stresses, consistent with the Fv/Fm results above. However, *SlHK2* RNAi plants grown under all stresses rose significantly in NPQ, when compared with WT plants. 

We further analyzed the transcript level of the photosynthesis genes (*PsbQ* and *PsbP*) on the 5-day drought, heat, and combined stress treatments using qRT-PCR. The transcript level of *PsbQ* differed slightly in the controlled conditions of *SlHK2* RNAi and WT plants. Conversely, the expression level of the *PsbP* gene remained unchanged. The relative expression levels of *PsbQ* and *PsbP* were much less reduced in *SlHK2* RNAi plants than in WT plants under all stressed conditions (Figure 4h,i).

### 3.3. Oxidative Stress Induced in SlHK2 RNAi and WT Plants under Drought, Heat, and Combined Stresses

**Figure 5:**
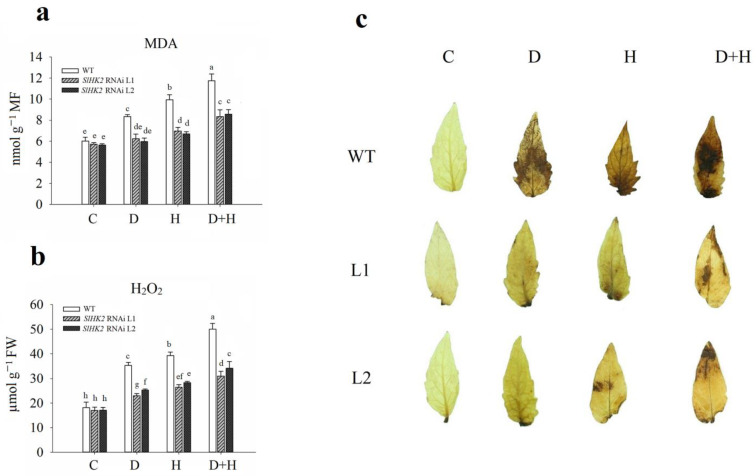
(**a**) Malondialdehyde content (MDA), (**b**) hydrogen peroxide (H_2_O_2_), and (**c**) histochemical staining results of H_2_O_2_ in WT and *SlHK2* RNAi plants after 5 days of drought (D), heat (H), and combined (D + H) stresses. For H_2_O_2_ staining, six leaves each of WT and *SlHK2* RNAi lines (L1 and L2) were used for each treatment. Values represent mean ± SE (n = 6). Different letters indicate significant differences among treatments at *p* < 0.05.

Immediately after the 5-day stress treatment, we measured the content of H_2_O_2_ and MDA in leaves of the drought-, heat-, and combined-stress treated *SlHK2* RNAi and WT plants. The MDA and H_2_O_2_ content under all stress treatments significantly increased in both *SlHK2* RNAi and WT plants, compared to unstressed plants (Figure 5a,b). Nevertheless, the negative impact of the combined stress was more evident than the individual drought stress and heat stress. The *SlHK2* RNAi plants exhibited less accumulation of MDA and H_2_O_2_ than did the WT plants following drought, heat, and combined stresses, indicating that alleviation of cell membrane damage occurred in *SlHK2* RNAi plants.

We also observed the endogenous accumulation of H_2_O_2_ in *SlHK2* RNAi and WT plant leaves by the DAB staining method under all stress conditions. After exposure to drought, heat, and combined stresses, the accumulation of H_2_O_2_ in the leaves of *SlHK2* RNAi and WT plants was remarkably higher than in the controlled plants (Figure 5c). The drought and heat stress treatments significantly induced the H_2_O_2_ accumulation in WT leaves, which increased further under combined stress. The accumulation of H_2_O_2_ in WT plants was significantly higher than that in *SlHK2* RNAi plants under all stress treatments. The lesser oxidative damage in *SlHK2* RNAi indicated high tolerance to abiotic stress.

### 3.4. Antioxidant Defense System in SlHK2 RNAi and WT Plants under Stress

**Figure 6:**
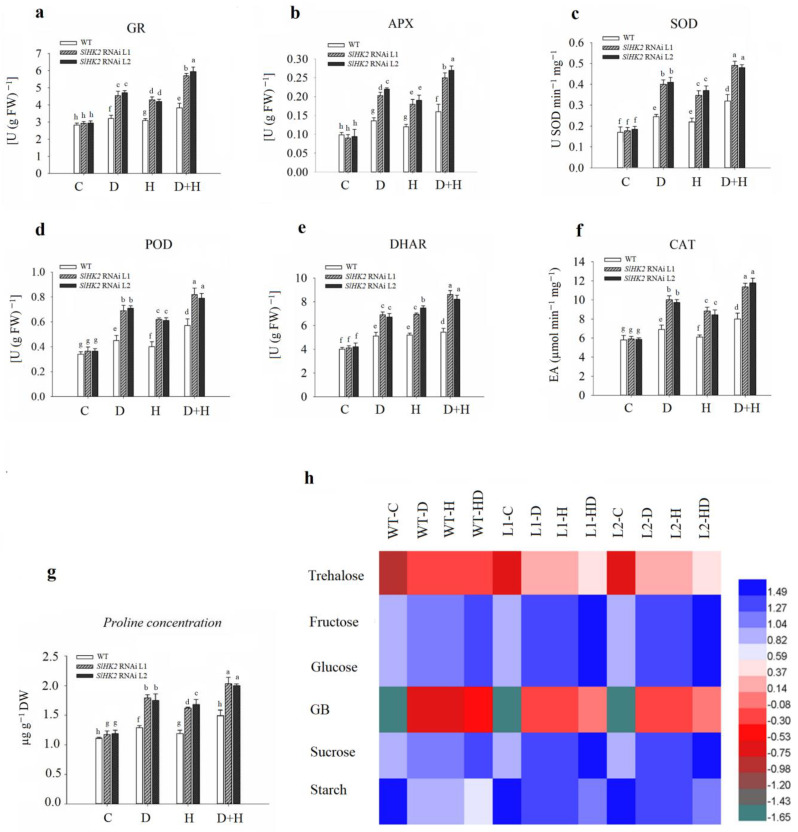
The activities of (**a**) glutathione reductase (GR), (**b**) ascorbate peroxidase APX, (**c**) superoxide dismutase (SOD), (**d**) peroxidase (POD), (**e**) dehydroascorbate reductase (DHAR), (**f**) catalase (CAT), (**g**) proline, and (**h**) osmoprotectants in leaves of the WT and *SlHK2* RNAi tomato plants under drought (D), heat (H) and combined (D + H) stresses. Blue represents a higher relative concentration, while green represents the lower relative concentration of the heat map in WT and *SlHK2* RNAi lines (L1 and L2). Values represent mean ± SE (n = 6). Different letters indicate significant differences among treatments at *p* < 0.05.

The antioxidant defense system in plants can scavenge free radicals produced through ROS following the abiotic stress environment. The activities of enzymatic antioxidants (including GR, APX, SOD, POD, DHAR, and CAT) were measured in *SlHK2* RNAi and WT plants after drought, heat, and combined stress treatments (Figure 6). GR, APX, SOD, POD, DHAR, and CAT activities increased in stressed WT and *SlHK2* RNAi plants compared to well-watered plants. Additionally, antioxidant activities were more induced in combined stress conditions than in individual drought and heat stress conditions. The *SlHK2* RNAi plants maintained much higher activities of all these antioxidants than did the WT plants in each stress scenario, supporting the conclusion that the enzymatic antioxidant defense system in *SlHK2* RNAi plants is substantially more active than in WT plants.

Osmoprotectants can detoxify ROS or mediate the stability of the enzymes implicated in the antioxidant defense system [1]. The accumulation of essential osmoprotectants (proline, glycine betaine (GB), trehalose, sucrose, glucose, fructose and, starch) was measured in drought, heat, and combined stress conditions in *SlHK2* RNAi and WT leaves. Drought, heat, and combined stresses increased the concentration of essential osmoprotectants in WT and *SlHK2* RNAi, as compared to plants grown under the control condition. The effect of combined stress on proline, GB, trehalose, sucrose, glucose, fructose, and sucrose concentration was greater than single-stress treatment (Figure 6g,h). The results showed a significant accumulation of osmoprotectants, such as proline, GB, trehalose, sucrose, glucose, fructose, and sucrose concentrations, after the fifth day of single drought, heat, and combined stresses in *SlHK2* RNAi plants, in contrast with WT plants. Additionally, starch contents were decreased in both *SlHK2* RNAi and WT plants in single and combined stresses. These results show that starch reduction was greater in the combined stress. In addition, the starch reduction level under each stress treatment was higher in WT plants than in *SlHK2* RNAi plants. Our results support the conclusion that in *SlHK2* RNAi plants, osmoprotectant accumulation is considerably more active than it is in WT plants.

**Figure 7:**
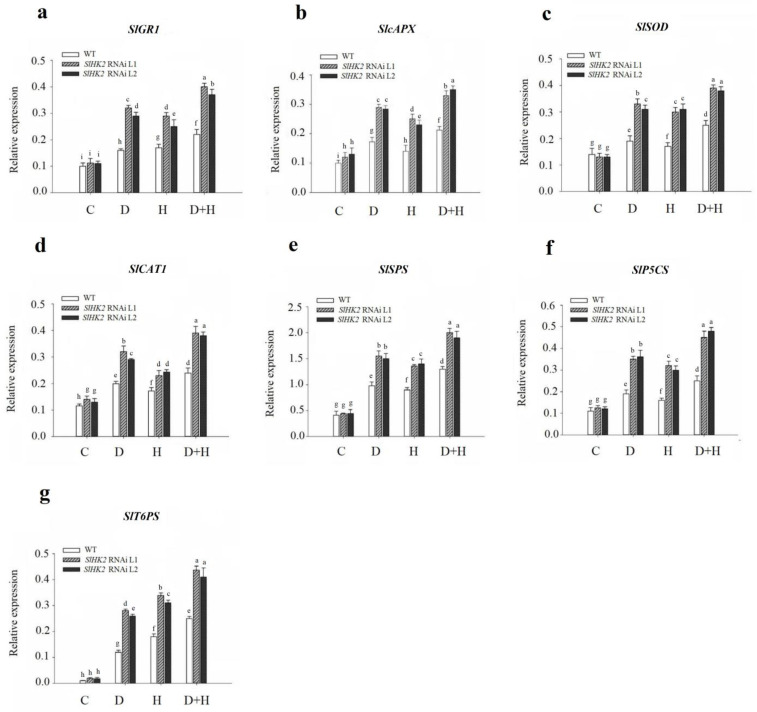
Transcript levels of (**a**) *SlGR1*, (**b**) *SclAPX*, (**c**) *SlSOD*, (**d**) *SlCAT1*, (**e**) *SlSPS*, (**f**) *SlP5CS*, and (**g**) *SlT6PS* genes, in leaves of *SlHK2* RNAi and WT tomato plants. Control, normal (hydrated) conditions (C); drought stress, 5 days no watering (D); heat stress, 5 days 32/26 °C day/night (H); combined drought and heat stress, 5 days no watering + 32/26 °C day/night for 5 days (D + HS). The relative expression level is determined by quantitative real-time PCR (using the *SlActin* gene as reference). Values represent mean ± SE (n = 6). Different letters show significant differences among treatments at *p* < 0.05.

Similarly, the gene expression level of antioxidant enzymes was estimated employing qRT-PCR (Figure 7). The expression level of *SlcAPX* and *SlCAT1* was higher in *SlHK2* RNAi plants than in WT plants under control conditions, while that of *SlGR1* and *SlSOD* exhibited no differences. Drought and heat stresses induced the highest gene expression of *SlGR1*, *SlcAPX*, *SlSOD,* and *SlCAT1* in *SlHK2* RNAi plants, compared to that of WT plants (Figure 7a–d). Notably, in *SlHK2* RNAi plants, the combination of drought and heat stresses provoked higher gene expression levels of *SlGR1*, *SlcAPX*, *SlSOD,* and *SlCAT1* than either stress individually. 

In addition, the transcript levels of sucrose (*SlSPS*), proline (*SlP5CS*), and trehalose *(SlT6PS)* synthesis genes were estimated in the *SlHK2* RNAi and WT plants. Under well-watered conditions, *SlSPS*, *SlP5CS,* and *SlT6PS* were not significantly different in WT and *SlHK2* RNAi plants. After five days, drought, heat, and combined stresses caused significant up-regulation of the osmoprotectant genes in *SlHK2* RNAi plants compared to WT plants (Figure 7e–g). The combined stress results showed that the transcript levels of these genes were much higher than they were for individual stresses. These results show that the up-regulation of antioxidant and osmoregulatory genes may contribute to *SlHK2* RNAi plants conferring abiotic stress tolerance.

## 4. Discussion

Previous studies revealed the function of cytokinin receptors, *AHK2*, as the negative regulators under a drought stress environment [21,23]. However, the function and significance of the *SlHK2* gene in response to individual drought and heat stresses, as well as combined stresses, in tomato plants are not yet clear. In the present research, we identified the precise role of *SlHK2* in abiotic stresses by exploring the significance of the loss-of-function on the acclimatization of tomato plants to these stresses. Our findings revealed that *SlHK2* acts as a negative regulator under individual drought stress, individual heat stress, and combined stress in tomato plants. Our study also demonstrated that suppressing the cytokinin function, and subsequently cytokinin signaling, is one of the approaches practiced by plants to survive against abiotic stress.

In our study, the drought-related genes were significantly up-regulated in *SlHK2* RNAi plants under drought and combined (drought + heat) stresses. RWC, electrolyte leakage, gas exchange parameters, and chlorophyll fluorescence parameters were less affected in *SlHK2* RNAi plants. Antioxidant enzyme activities, genes encoding antioxidant enzyme, osmoprotectant accumulation, and osmoprotectant gene expression were significantly induced in *SlHK2* RNAi plants. Additionally, MDA and H_2_O_2_ were less accumulated in *SlHK2* RNAi plants under all these stress conditions.

Previous research on drought stress responses with *Arabidopsis* CK-deficient mutants, such as *ahk2*, *ahk3*, and *ahk2,3* indicated that CKs, and therefore CK signaling, can act as negative regulators of plant drought adaptation. These outcomes indicated that mechanisms happen in *ahk2*, *ahk3*, and *ahk2,3 Arabidopsis* plants that cause suppression of CK signaling in drought stress and osmotic stress, leading to a series of changes and, therefore, biochemical and physiological adjustments for plant endurance [22,23,40]. Abiotic stress treatments were shown to suppress the expression of *SlHK2*, *SlRR1*, *SlRR3*, and *SlRR5* genes in plants, confirming the negative regulatory roles of *SlHK2* under these stresses (Figure S2). This research reveals that plants may down-regulate *SlHK2*, *SlRR1*, *SlRR3*, and *SlRR5* genes, and therefore the cytokinin response increases the survival upon adverse conditions. Previous research findings showed that plants can initiate acclimatizing mechanisms to suppress CK signaling by down-regulating CK signaling genes’ expression, to cope with abiotic stress [21,41].

Under abiotic stress treatments, *SlHK2* RNAi plants sustained higher relative water content, which may strengthen their capacity to harbor membrane structure and increase responsiveness to ABA, ultimately enhancing the tolerance of tomato plants to these stresses (Figure 2a). The same trend was observed in cytokinin *arr1,10,12-* and *ahp2,3,5*-tolerant plants because of their ability to carry a higher relative water status under drought stress. The higher water content is correlated with enhanced membrane structure protection, as shown by lesser ion leakage [21,42].

*SlHK2* RNAi plants increase the strength of membrane structures, as evidenced by a lower electrolyte leakage percentage in abiotic stress conditions (Figure 2b). *SlHK2* RNAi plants direct the up-regulation of osmolyte biosynthesis-related sucrose (*SlSPS)*, proline (*SlP5CS*), and trehalose (*SlT6PS*) genes to activate defense mechanisms as well as enhance the stability and repair of membrane structures, and to sustain osmotic adjustments throughout drought, heat, and combined stresses that improve the performance of the plants (Figure 7e–g). Previous studies revealed that cytokinin loss-of-function *Arabidopsis* plants improved drought tolerance through up-regulation of osmolyte synthesis-related genes [21]. In plants, proline acts as an osmoprotectant and enables plants to tolerate abiotic stress conditions [43]. The individual and combined stresses (drought and heat) exhibited significant proline accumulation in *SlHK2* RNAi plants (Figure 6g), which is consistent with research [9] that confirmed that higher levels of proline are required in plant protection in conditions of drought stress and heat stress. Several previous studies also support our findings that proline accumulation occurs in resistant plants subjected to stress conditions [44,45], because of its attribute of maintaining membrane structures and scavenging ROS [46]. Overall, our findings indicate that the strengthening of membrane structures and osmoprotectant accumulation performs an important part of *SlHK2* RNAi plants’ adaptation to environmental stresses.

Previous research revealed that the up-regulation of cuticular wax genes decreases electrolyte leakage in plants under dehydration conditions [39]. In addition, we observed that various genes implicated in regulating cuticular wax were significantly up-regulated in *SlHK2* RNAi plants, compared to WT plants (Figure 3). Furthermore, our outcomes indicated that total wax loads in *SlHK2* RNAi plants were significantly enhanced, in comparison with WT plants, in normal growth conditions (Figure 3e). The initial research showed that wax accumulation decreases electrolyte leakage in *arr1,10,12* plants by initiating the biosynthesis of cuticular wax [21]. Another previous study confirmed that in resistant *ahp2,3,5* plants, less electrolyte leakage was perceived due to increasing cuticular wax accumulation [41]. Our finding proposed that drought stress-, heat stress-, and (especially) combined-stress tolerance and reduced electrolyte leakage perceived in *SlHK2* RNAi plants could be associated with increased cuticular wax accumulation. In addition, these outcomes suggest that the accumulation of cuticular wax might be directed via cytokinins, inclusive of a critical function provided by *SlHK2* in adverse conditions.

Stomata play an important role in controlling gas exchange and water evaporation in plants. The primary response to abiotic stresses is a change in the stomatal conductance [47]. However, reducing stomatal conductance response inhibits plant growth, as it limits CO_2_ and thus impairs the photosynthesis process. A compromise between water transpiration and carbon assimilation must be reached for optimal growth under stress. In this study, *SlHK2* RNAi plants sustained a higher stomatal conductance than did WT plants under individual drought stress, individual heat stress, and combined stress conditions. Evidently, this effective stress-avoidance mechanism could be partially ascribed to the improved root system. The root parameters, including the length and depth of roots, have been associated with stress tolerance in various species and are supposed to contribute to plant performance under abiotic stress environments [48,49,50]. Plants with more extensive root systems are better tolerant of drought conditions, which may be provoked partially by the role of cytokinins in regulating the response to stresses [21,38,51]. Previous research indicated that root development is required for plants to be able to sense the availability of water [52]. It could be that *SlHK2* RNAi increases the root growth of plants, enabling this practice and improving access to water, and positively regulating stomatal conductance under abiotic stresses.

Previous studies have revealed that the increase in GB and trehalose accumulation leads to an improved photosynthesis function in plants [1]. Our study showed that the photosynthesis process is protected in *SlHK2* RNAi plants under single and combined stresses (Figure 4). The protection of photosynthesis machinery may be associated with increased GB and trehalose activities in *SlHK2* RNAi plants. Prior research was consistent with our findings, reporting that GB and trehalose might play significant roles in plants’ tolerance to abiotic stress environments due to their ability to stabilize photosynthesis functions [10]. In addition, these osmoprotectants protected the structures of membranes and thylakoids against abiotic stress instability [53,54]. Overall, this protection of photosynthetic ability may enable *SlHK2* RNAi plants to overcome these stress conditions better than WT plants are able to do.

### Oxidative Stress

Reactive oxygen species (ROS), which are principally comprised of O^2−^ and H_2_O_2_, function as signaling molecules for the induction of plant responses upon numerous abiotic stress conditions [10,55,56]. A higher level of ROS eventually results in cell damage and oxidative stress in the absence of prompt scavenging through antioxidant enzymes. In our study, under all stress conditions, *SlHK2* RNAi plants showed less accumulation of H_2_O_2_ contents and MDA levels, indicating that *SlHK2* RNAi plants protect membrane structures by repressing lipid peroxidation in stress conditions, probably through the removal of ROS (Figure 5).

In addition, cell membrane instability and membrane injury provoked by oxidative stress is used as a basis to evaluate a plant’s level of abiotic stress tolerance [57,58]. Higher activities of ROS scavenging enzymes protect plant cells from death caused by oxidative injury. The anti-oxidation mechanism is crucial for plant tolerance and involves SOD, APX, CAT, and POD. APX, because of its higher affinity for H_2_O_2_ when compared with CAT, executes a decisive function in a plant’s protection against oxidative stress [14,59,60]. Previous studies revealed that APX and SOD activities were higher in tomato plants during heat and dehydration conditions. The significant induction of protecting antioxidant enzymes in *SlHK2* RNAi plants might be attributed to the plants’ improved tolerance toward the drought stresses, heat stresses, and (especially) combined stresses (Figure 6). Therefore, our study suggests an important role for *SlHK2* in protecting plants from abiotic stress environments.

## 5. Conclusions

This study demonstrated the evolutionary divergence of the role of *SlHK2* in stress responses and provided valuable information for engineering stress-resistant crops. In addition, photosynthesis machinery protection, membrane stability, osmoprotectant accumulation, and cuticular wax could play decisive roles in conferring enhanced tolerance of *SlHK2* RNAi plants to drought, heat, and combined stresses. The findings also unveiled an innovative research basis for developing plants with increased tolerance to abiotic stress combinations, to minimize yield losses associated with global climate change.

## Data Availability

Not applicable.

## Supplementary Materials

The following supporting information can be downloaded at: https://www.mdpi.com/article/10.3390/plants11020154/s1, Figure S1: Schematic representation of the used plasmid construct of SlHK2 in tomato plants; Figure S2: The transcript levels of *SlHK2* (a)*, SlRR1* (b)*, SlRR3* (c) and *SlRR5* (d) in leaves of WT and *SlHK2* RNAi plants grown under well-watered (C), drought (D), heat (H) and combined stress (D + H) conditions; Figure S3: GUS staining of fruit (a), leaf (b) and sepal (c) in *SlHK2* RNAi plants under well-watered conditions; Table S1: Primers used in this study; Table S2: The root length of *SlHK2* RNAi and WT plants.

## Abbreviations

APX: ascorbate peroxidase; BSTFA, N, O-(trimethylsilyl); CAT, catalase; DHAR, dehydroascorbate reductase; GC-MS, gas chromatography-mass spectrometry; GC-FID, gas chromatography with flame ionization detection; GR, glutation reductase; MDA, malondialdehyde; POD, peroxidase; qRT-PCR, quantitative real-time PCR; ROS, reactive oxygen species; RWC, relative water content; SOD, superoxide dismutase; WT, wild-type.
