# Supplementary material for: Down-Regulation of Cytokinin Receptor Gene SlHK2 Improves Plant Tolerance to Drought, Heat, and Combined Stresses in Tomato
Source: Plants (Basel). 2022 Jan 7;11(2):154. doi: 10.3390/plants11020154 (PMC8779561; doi:10.3390/plants11020154)
Supplement: Supplementary file 1 [file plants-11-00154-s001.zip › plants-1471562-supplementary.pdf]

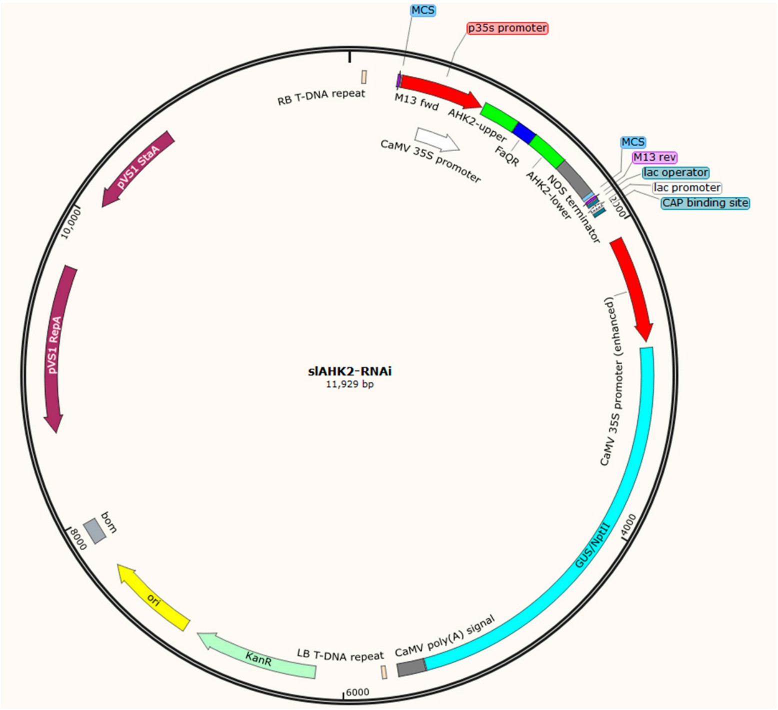

Figure S1. Schematic representation of the used plasmid construct of SlHK2 in tomato plants. The reporter gene *GUS* was included with the plasmid p35S:SlHK2-RNAi, under the control of the CaMV35S promoter.

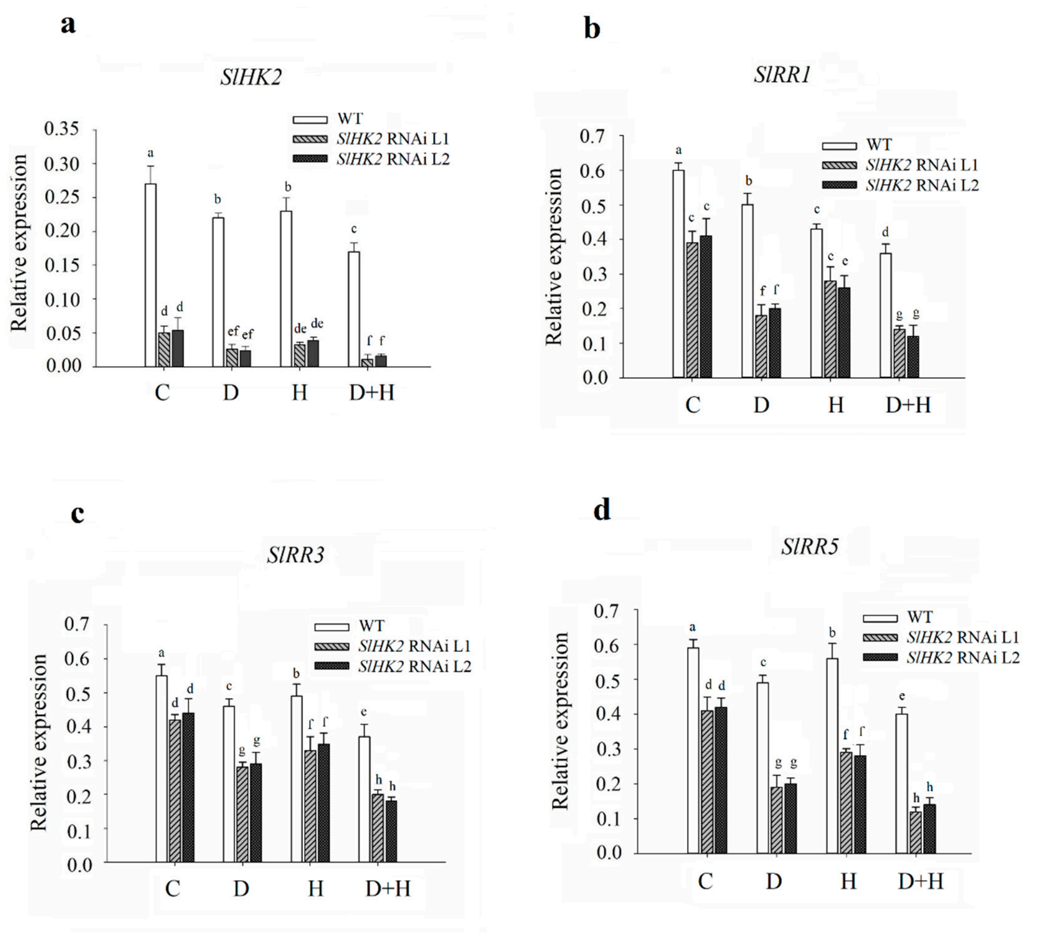

Figure S2. The transcript levels of *SlHK2* (a), *SlRR1* (b), *SlRR3* (c) and *SlRR5* (d) in leaves of WT and *SlHK2* RNAi plants grown under well-watered (C), drought (D), heat (H) and combined stress (D+H) conditions. Values represent mean ± SE (n = 6). The relative expression levels were determined by quantitative real-time PCR using the *Actin* gene as reference). Different letters indicate significant differences at $p < 0.05$ among treatment.

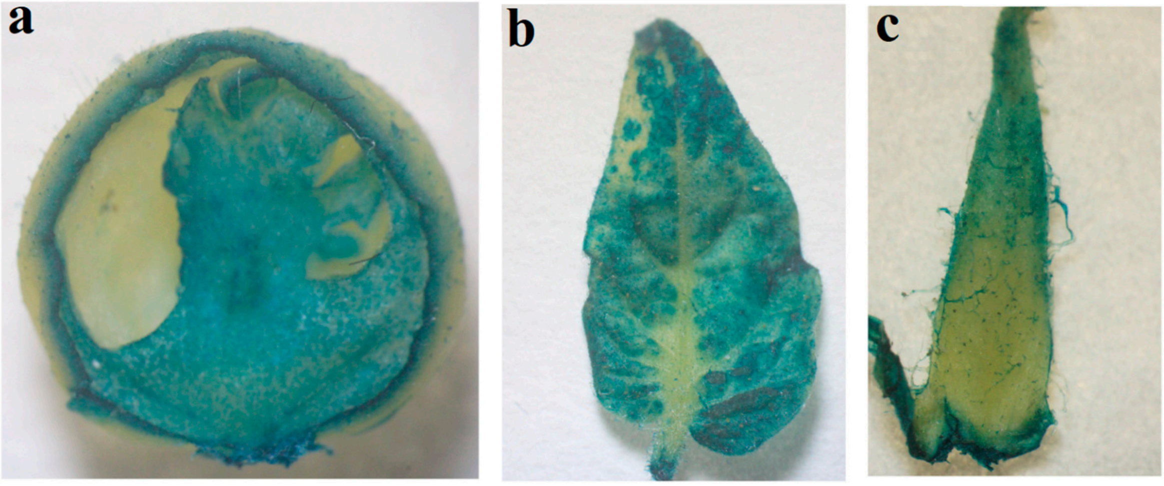

Figure S3. GUS staining of fruit (a), leaf (b) and sepal (c) in SlHK2 RNAi plants under well-watered conditions. The staining was performed on the 55-day old *SlHK2* RNAi plant's, whereas the sepal and fruit samples were stained after 18 days of DAF. Values represent mean ± SE (n = 6). Different letters indicate significant differences at $p < 0.05$ among treatment.

Table S1. Primers used in this study.

Gene	Forward	Reverse
SlActin	CTTGTCTGTGACAATGGAACTG	ATACCCACCATCACACCAGTAT
SlHK2	GGGATTGAGTTGGCTGTTTATG	TGCCCTTTGTCATTTGTGAAC
SlRR1	GGAGGAAGGTGCTGAAGAAT	CCGTTTCTTTGTTGTCATCCC
SlRR3	GAAGATGGTCTGAGGGCTTTAG	CCAGTCATTCCTGGCATACAA
SlRR5	TCTAGCTGTTGATGACAACCTTATT	CCAACGCCCTTAGACCATTT
SlDREB1	TGCACCGTTGCTTCTGGTTTCG	ACTGAGCTCAATGGTGTGTCAGC
SlNCED	CCGGTGGTTTACGACAAGAA	TCCAGAGGTGGAAACAGAAAC
SlHsp17.4	TCGTTGTGGATATGCCAGGGTTGA	TCTCCGGCAGACTAAACT
SlHsp21	ATGTCACCGATGAGAACAATGCGG	TCATGAATGTCCCATGGAGTGCGT
SlGR1	TTGGTGGAACGTGTGTTCTT	TCTCATTCACTTCCCATCCA
SlcAPX	TCTGAATTGGGATTTGCTGA	CGTCTAACGTAGCTGCCAAA
SlCAT1	TGATCGCGAGAAGATACCTG	CTTCCACGTTCATGGACAAC
SlSOD	AGAAAGCTGTTGCTGTCCTTA	CCAGGAGCAAGTCCAGTTATAC
SlP5CS	CCCACACCGATAGCATCATTAC	TCCAAAGCGGAATCCATCAC
LeSPS	GATGAGTGAGATGGGGGAGA	TCTTGGTCCAAAAGGAATGC
LeT6PS	TGACAAACAGCAGGCTCATC	CTTCTTGACTTGGGGCTTTG
PsbQ	CGCTTGAGTACTGTTAGCACCA	AATGGCAAACTGAAGTCCCT

PsbP	AAAATTATATCACTCCGTGTC	GTATCTGAGAAGTCATCAGC
SlKCS1	ATATCACTGGCATCACCACGAG	CGTAACCAATAAATGCAGTGCCT
SlCER3	ATATCACTGGCATCACCACGAG	CGTAACCAATAAATGCAGTGCCT
SlLTP1	CTTGTCTGTGACAATGGAACTG	ATACCCACCATCACACCAGTAT
SlSTTS1	ACAGGCAACTGATGGTCATT	CCATCTTCGTTCTGATGACAG

Table S2. The root length of SlHK2 RNAi and WT plants.

WT	*SlHK2* RNAi L1	*SlHK2* RNAi L2
15 ± 1	24 ± 2	25 ± 1
18 ± 1	27 ± 3	27 ± 2
14 ± 3	23 ± 2	22.4 ± 1
